# The antithrombin activity recovery after substitution therapy is associated with improved 28-day mortality in patients with sepsis-associated disseminated intravascular coagulation

**DOI:** 10.1186/s12959-023-00556-6

**Published:** 2023-11-02

**Authors:** Toshiaki Iba, Tomoki Tanigawa, Hideo Wada, Jerrold H. Levy

**Affiliations:** 1 Department of Emergency and Disaster Medicine, Juntendo University Graduate School of Medicine, 2-1-1 Hongo Bunkyo-Ku, Tokyo, 113-8421 Japan; 2 Medical Affairs Section, Research & Development Division, Japan Blood Products Organization, 15F Tamachi Station Tower N 3-1-1 Shibaura, Minato-Ku, Tokyo, Japan; 3 Department of General and Laboratory Medicine, Mie Prefectural General Medical Center, Yokkaichi, Japan; 4 Department of Anesthesiology, Critical Care, and Surgery, Duke University School of Medicine, Durham, NC USA

**Keywords:** Antithrombin, Disseminated intravascular coagulation, Sepsis, Organ failure, Sequential organ failure assessment

## Abstract

**Background:**

Disseminated intravascular coagulation (DIC) is a common and critical complication in sepsis. Antithrombin activity, which is considered a biomarker for disease severity, was measured in septic DIC treated with antithrombin concentrates in this study.

**Methods:**

We conducted a retrospective analysis of post-marketing survey data that included 1,800 patients with sepsis-associated DIC and antithrombin activity of 70% or less who were treated with antithrombin concentrates. The changes in sequential organ failure assessment (SOFA) score, DIC score, and antithrombin activity were sequentially assessed. Logistic regression analysis and receiver operating characteristic (ROC) curve analysis were performed to evaluate the performance of antithrombin activity to assess 28-day survival. Furthermore, the relationship between post-treatment antithrombin activity and survival was examined by Logistic regression analysis.

**Results:**

Sex, baseline SOFA score, baseline antithrombin activities, and the presence of pneumonia and soft tissue infection were significantly associated with 28-day mortality. The area under the curve for mortality was 0.639 for post-treatment antithrombin activity, and higher than those of baseline- and delta antithrombin activities. Logistic regression analysis revealed that higher post-treatment antithrombin activity was associated with better 28-day survival. When post-treatment antithrombin activity was more than 80%, the estimated survival was 88.2%. Whereas, the survival was 74.4% when the antithrombin activity was 80% or less (*P* < 0.0001). However, the relationship between post-treatment antithrombin activity and 28-day survival was considerably different between patients who recovered from DIC by Day 6 compared to those who did not. Similarly, the estimated 28-day survival, based on antithrombin activity, varied among patients with high and low SOFA scores, and the calculation needs to be adjusted based on the severity of the condition.

**Conclusions:**

Post-treatment antithrombin activity measurement was helpful in estimating the 28-day survival in patients with sepsis-associated DIC. However, patient outcomes vary considerably depending on factors that include baseline SOFA score, age, and baseline antithrombin activity. These variables play a substantial role in determining patient prognosis and should be considered when evaluating and interpreting the results.

**Supplementary Information:**

The online version contains supplementary material available at 10.1186/s12959-023-00556-6.

## Background

Disseminated intravascular coagulation (DIC) is a common and critical complication in sepsis, and the mortality increases considerably when septic patients develop DIC. Gando et al. [1] reported that the prevalence of DIC in patients with severe sepsis and treated in ICU was 50.9%, and the mortality rate was 24.8%, significantly higher than that in patients without DIC (17.5%). DIC in sepsis is characterized by activated coagulation, suppressed fibrinolysis, and endothelial injury, which all contribute to thromboinflammation [2, 3]. Since DIC is an important factor that determines the clinical course of septic patients, early evaluation of the severity is important to initiate appropriate interventions [4].

Antithrombin is the most abundant and crucial physiological anticoagulant [5]. However, antithrombin levels are known to decrease markedly in sepsis-associated DIC [6], and its activity is an important biomarker reflecting sepsis-associated DIC severity [7]. Beyond its use as a diagnostic biomarker, decreased antithrombin levels are also a therapeutic target when antithrombin supplementation occurs, especially in Japan and based on guidelines [8]. Subsequently, besides the use of recombinant thrombomodulin, anticoagulation with antithrombin concentrates is recommended for patients with septic DIC with antithrombin levels of 70% or less by the Japanese guidelines for sepsis management [9]. The limiting factor in this therapy is the optimal dose has not been determined, and more work is needed to better determine the optimal target antithrombin activity.

Post-repletion antithrombin activity is also a prognostic marker. Previous studies have demonstrated that antithrombin activity after three consecutive days of antithrombin substitution estimated the morbidity and mortality in sepsis-associated DIC [10]. Akahoshi et al. [11] reported the survival rates of septic DIC were significantly higher in patients who achieved post-treatment antithrombin activity of more than 70% or 80%. However, these studies have examined the relationship between antithrombin activity and mortality using receiver operating characteristic (ROC) curve analysis and compared the mortality rates above and below cutoff values. In the present study, our goal was to better determine post-treatment antithrombin activity and mortality by dealing with antithrombin activity as continuous variables in a larger cohort.

## Materials and methods

### Patient selection and data collection

**Fig. 1:**
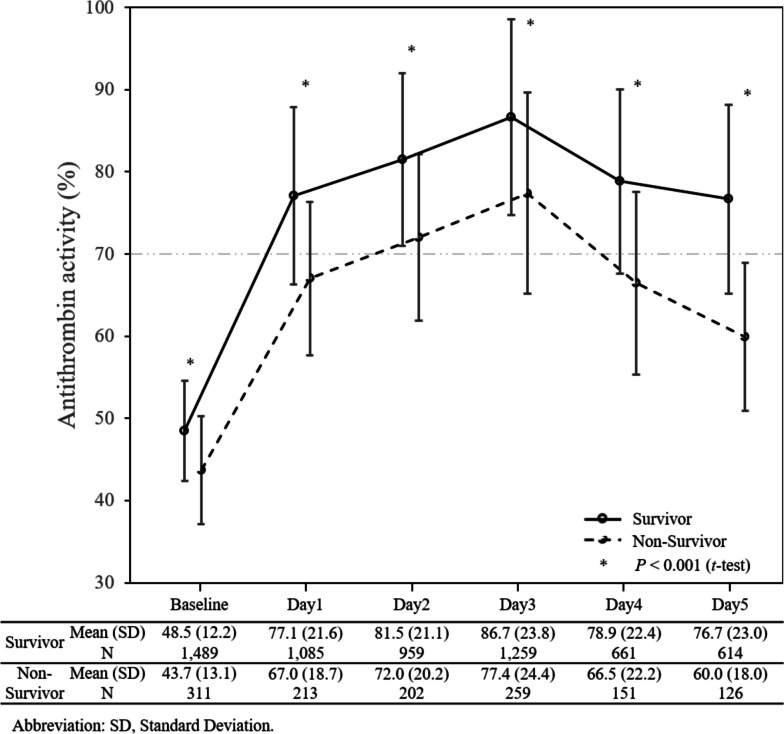
The changes in antithrombin activity. Changes in the mean antithrombin activity ± standard deviation in the survivor and non-survivor groups are plotted. The antithrombin activity increased to above 80% after the treatment in the survivor group, but not in the non-survivor group

One thousand eight hundredpatients with sepsis-associated DIC with antithrombin activity of 70% or less and treated with antithrombin concentrate between April 2013 and April 2016 were retrospectively analyzed. The study excluded patients allergic to antithrombin, patients with leukemia, malignant tumors, liver cirrhosis, and patients after cardiopulmonary arrest (Supple. Figure 1). DIC was diagnosed with the Japanese Association for Acute Medicine (JAAM) DIC criteria [12], and the serial data of the DIC score and SOFA score were collected on the day before antithrombin treatment (Day 0) to Day 6. Antithrombin activity was measured daily from Day 0 to Day 5, and the post-treatment antithrombin activity was defined as the day after the end of the antithrombin dosing period. DIC was judged as resolved when the DIC score decreased to less than 4 by Day 6. The data from the patients who died before Day 6 were included in the analysis. Therefore, patients whose DIC had not improved by the time of death were recorded "DIC not resolved." However, the data for the patients after their decease were recorded as blank or not available for calculation. Missing data on post-treatment antithrombin activity was obtained by the Last Observation Carried Forward method, and 211 cases were recruited. The patient data are from the post-marketing surveillance conducted by Japan Blood Products Organization at 277 hospitals in Japan. The study was reviewed by the Institutional Review Board of each hospital and was conducted in accordance with the Declaration of Helsinki and Good Post-Marketing Study Practice. Because of the anonymous nature of the data, the requirement for informed consent was waived.

### Intervention and treatment

Antithrombin concentrate (Neuart®; Japan Blood Products Organization, Tokyo) was administered on a daily basis to patients who met DIC criteria. Antithrombin was administered through drip infusion over several hours during the daytime, and sample collection was conducted the following morning. There was no limitation in the duration of antithrombin treatment, and each physician decided based on clinical judgment. Antithrombin doses were selected based on the doctor’s decision, but since the Japanese healthcare system approves the antithrombin dose of 1,500 IU/day, most cases received the recommended dose. There were also no restrictions on the concomitant use of heparin, protease inhibitors (nafamostat mesilate, gabexate mesilate), recombinant thrombomodulin, or other anticoagulants.

### Statistical analysis

Baseline characteristics were compared between survivors and non-survivors. Continuous variables were checked for normality (kurtosis, skewness, difference between mean and median), and then T-tests were performed. Categorical variables were presented as frequency distributions and percentages, and Fisher's exact test was performed. Receiver operating characteristic (ROC) curve analysis was performed for antithrombin activity at the respective points (baseline, post-treatment, and differences between them) and compared their ability to predict 28-day mortality by evaluating the area under the curve (AUC). In addition, the Youden index was used to calculate the optimal cutoff value. Univariate logistic regression was performed to examine the risk for survival up to day 28. The reference was the first category for categorical variables. Considering the correlation and collinearity between each variable, the first multivariate logistic regression was conducted to narrow down the variables. The variable selection method was Stepwise, with α = 0.25 as the inclusion criterion and α = 0.15 as the exclusion criterion. Based on these results, a final multivariate logistic regression was conducted. The relationship between post-treatment antithrombin activity as a continuous variable and 28-day survival was estimated by logistic regression analysis. In addition, a logistic regression analysis stratified by risk factors. Results are reported as odds ratio (OR), 95% confidence interval (CI), and *P* values. All statistical analyses were performed using SAS (ver. 9.4, SAS Institute, Co., Ltd., Cary, NC, USA) and R (version 4.1.1, R Foundation for Statistical Computing, Vienna, Austria).

## Results

The patients’ demographics are summarized in Table 1. Among the 1,800 patients, 1,489 survived (82.7%), while 311 (17.3%) died. The proportion of females was significantly higher among the survivors (*P* < 0.001). The median age of the survivors was 73 years, and that of the non-survivors was 76 years (*P* < 0.001). The mean SOFA score was significantly lower in survivors (8.9 vs. 11.7, *P* < 0.001), while the JAAM/DIC score was not different between survivors and non-survivors. The mean baseline antithrombin activity was higher in survivors (48.5% vs. 43.7%, *P* < 0.0001). Pneumonia was less common in survivors (*P* < 0.001).

**Table 1:** Baseline characteristics of the patients

Factor		Survivor		Non-survivor		Test
		1,489		311		
Sex	Male	849	57.0 (%)	210	67.5 (%)	*P* = 0.0006
	Female	640	43.0 (%)	101	32.5 (%)	
Age (y.o.)	n	1,489		311		*P* < 0.0001
	Mean S.D	70	15.5	73.6	13.5	
	Median	73		76		
Body weight (kg)	n	1,472		303		*P* = 0.5389
	Mean S.D	55.4	13	55.9	14.6	
	Median	54.4		55		
SOFA score	n	1,298		264		*P* < 0.0001
	Mean S.D	8.9	3.7	11.7	3.9	
	Median	9		12		
JAAM DIC score	n	1,489		311		*P* = 0.7520
	Mean S.D	5.6	1.4	5.6	1.3	
	Median	5		5		
Baseline antithrombin activity	n	1,489		311		*P* < 0.0001
	Mean S.D	48.5	12.2	43.7	13.1	
	Median	50		43.4		
Source of infection	Pneumonia	269	18.1 (%)	117	37.6 (%)	*P* < 0.0001
	Digestive tract	377	25.3 (%)	65	20.9 (%)	*P* = 0.1109
	Pyelonephritis	215	14.4 (%)	20	6.4 (%)	*P* < 0.0001
	Biliary tract	138	9.3 (%)	20	6.4 (%)	*P* = 0.1227
	Skin/soft tissue	98	6.6 (%)	33	10.6 (%)	*P* = 0.0162
	Urinary tract	35	2.4 (%)	3	1.0 (%)	*P* = 0.1891
	Intravenous catheter	54	3.6 (%)	7	2.3 (%)	*P* = 0.2999
	Others	92	6.2 (%)	15	4.8 (%)	*P* = 0.4291
	unknown	187	12.6 (%)	72	23.2 (%)	*P* < 0.0001
Co-administration	Heparin	236	15.8 (%)	56	18.0 (%)	*P* = 0.3527
	Protease inhibitor	306	20.6 (%)	61	19.6 (%)	*P* = 0.7572
	Thrombomodulin	871	58.5 (%)	189	60.8 (%)	*P* = 0.4860
Daily dose of antithrombin concentrate (IU)	n	1,489		311		*P* = 0.5948
	Mean S.D	1571.6	450.5	1586.4	424.9	
	Median	1500		1500		
Duration of antithrombin concentrate (day)	n	1,489		311		*P* = 0.0143
	Mean S.D	3.2	1.9	3.5	2.2	
	Median	3		3		
Total dose of antithrombin concentrate (IU)	n	1,489		311		*P* < 0.0001
	Mean S.D	4736.8	1905.2	5308.3	2311.1	
	Median	4500		4500		

*Abbreviations*: *S.D* Standard deviation, *SOFA* Sequential organ failure assessment, *JAAM DIC* Japanese association for acute medicine disseminated intravascular coagulation

The changes in antithrombin activity are shown in Fig. 1. The mean baseline activity was below 50% in both survivors and non-survivors. In both groups, the activity increased after the substitution of antithrombin and peaked at Day 3. The mean antithrombin activity exceeded 80% in survivors but did not reach the level in non-survivors (*P* < 0.001).

Univariate logistic regression analysis identified that age, baseline SOFA score, baseline antithrombin activity, presence of pneumonia, and presence of pyelonephritis, and presence of skin/soft tissue infection were the significant factors that influenced survival (Table 2). The following multivariate logistic regression analysis demonstrated age, baseline SOFA score, and the presence of pneumonia or skin/soft tissue infection as significant factors associated with worsened survival. On the other hand, baseline antithrombin activity was found to be associated with better survival (Table 3).

**Table 2:** Univariate logistic regression analysis for survival factors

	OR	95%CI			*P* value
Sex = male	1.567	1.213-2.036			0.0007
Age (y.o.)	1.018	1.009-1.027			0.0002
Body weight (kg)	1.003	0.994-1.012			0.5077
Baseline SOFA score	1.218	1.174-1.265			< 0.0001
Baseline JAAM DIC score	1.015	0.926-1.110			0.7519
Baseline antithrombin activity	0.738	0.666-0.816			< 0.0001
Pneumonia	2.735	2.096-3.561			< 0.0001
Digestive tract	0.779	0.575-1.043			0.1003
Pyelonephritis	0.407	0.246-0.639			0.0002
Biliary tract	0.673	0.402-1.069			0.1099
Skin/soft tissue	1.685	1.098-2.525			0.0137
Urinary tract	0.405	0.097-1.132			0.1345
Intravenous catheter	0.612	0.252-1.270			0.2271
Others	0.769	0.423-1.307			0.3591
Unknown	2.098	1.540-2.835			< 0.0001
Heparin	1.166	0.840-1.597			0.3484
Protease inhibitor	0.943	0.689-1.275			0.7093
Thrombomodulin	1.099	0.857-1.414			0.4582

*Abbreviations*: *OR* Odds ratio, *CI* Confidence interval, *SOFA* Sequential organ failure assessment, *JAAM DIC* Japanese association for acute medicine disseminated intravascular coagulation

**Table 3:** Multivariate logistic regression analysis for survival factors

	OR	95%CI			*P* value
Sex = male	1.270	0.937-1.730			0.1266
Age (y.o.)	1.022	1.011-1.034			0.0001
Baseline SOFA score	1.197	1.151-1.247			< 0.0001
Baseline antithrombin activity	0.806	0.713-0.909			0.0005
Pneumonia	2.256	1.646-3.087			< 0.0001
Pyelonephritis	0.572	0.330-0.946			0.0368
Biliary tract	0.478	0.237-0.891			0.0280
Skin/soft tissue	2.030	1.218-3.308			0.0053

*Abbreviations*: *OR* Odds ratio, *CI* Confidence interval, *SOFA* Sequential organ failure assessment

**Fig. 2:**
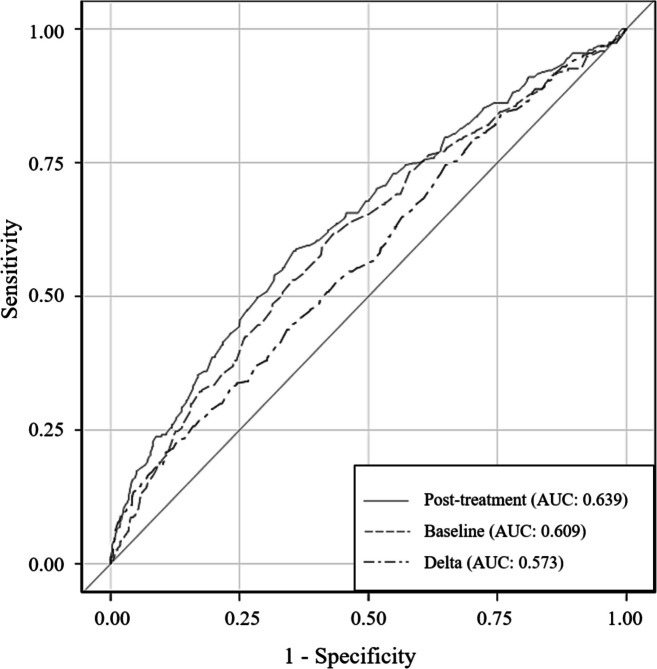
Comparison of the receiver operating characteristic curves of the antithrombin activities for 28-day survival. Receiver operating characteristic (ROC) curves comparing post-treatment antithrombin activity (solid line), baseline antithrombin activity (dashed line), and delta antithrombin activity (post-treatment activity minus baseline activity, dashed-dotted line) for 28-day survival are depicted. The respective areas under the ROC curves (AUCs) for these three activities were 0.639, 0.609, and 0.573, and the cutoff values were 80%, 47%, and 16.4%

Figure 2 shows the ROC curves of the baseline, post-treatment, and delta antithrombin activity for survival. The AUCs of the three antithrombin activities were 0.609, 0.639, and 0.573, and the post-treatment activity showed the best performance. The cutoff values of each antithrombin activity were 47%, 80%, and 16.4%, respectively.

**Fig. 3:**
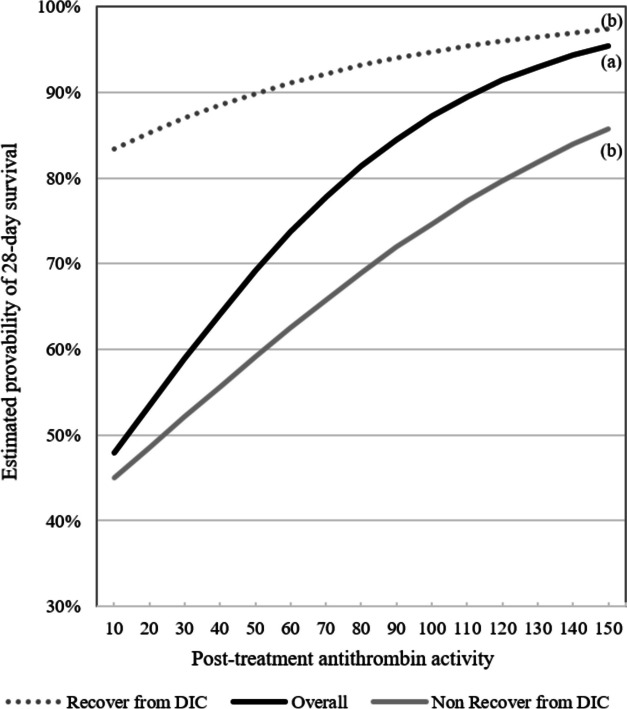
Estimated probability of 28-day survival rate for post-treatment antithrombin activity. Logistic regression analysis revealed a significant association between the post-treatment antithrombin and the 28-day mortality (*P* < 0.001) (**a**). The estimated probabilities of the 28-day survival rate are shown based on the recovery timing from disseminated intravascular coagulation (DIC). In the group that achieved recovery by Day 6, the estimated probabilities of survival were higher compared to the group that did not achieve recovery by Day 6 (odds ratio: 6.13, 95% confidence interval: 4.51–8.34) (**b**)

The estimated probability of a 28-day survival rate for post-treatment antithrombin activity was constructed by the logistic regression model (Fig. 3). When post-treatment antithrombin activities were 60%, 80%, and 100%, the survival rates were 74% (95%CI: 0.70–0.77), 81% (0.79–0.83), and 87% (0.85–0.89), respectively (a). Significant differences in survival rates were observed when the patients were categorized into two groups: those who achieved early recovery from DIC by Day 6 and those who did not recover by Day 6. When antithrombin activity after treatment was 60%, 80%, and 100%, in the early recovery group, survival rates were 91% (95% CI: 0.88–0.93), 93% (0.91–0.95), and 95% (0.93–0.96), respectively. Meanwhile, in the group where recovery was not achieved, survival rates were 63% (95% CI: 0.58–0.67), 69% (0.66–0.72), and 75% (0.71–0.78), respectively (Suppl. Tab. 1). The difference in survival rates is particularly pronounced at lower levels of antithrombin activity (b).

**Fig. 4:**
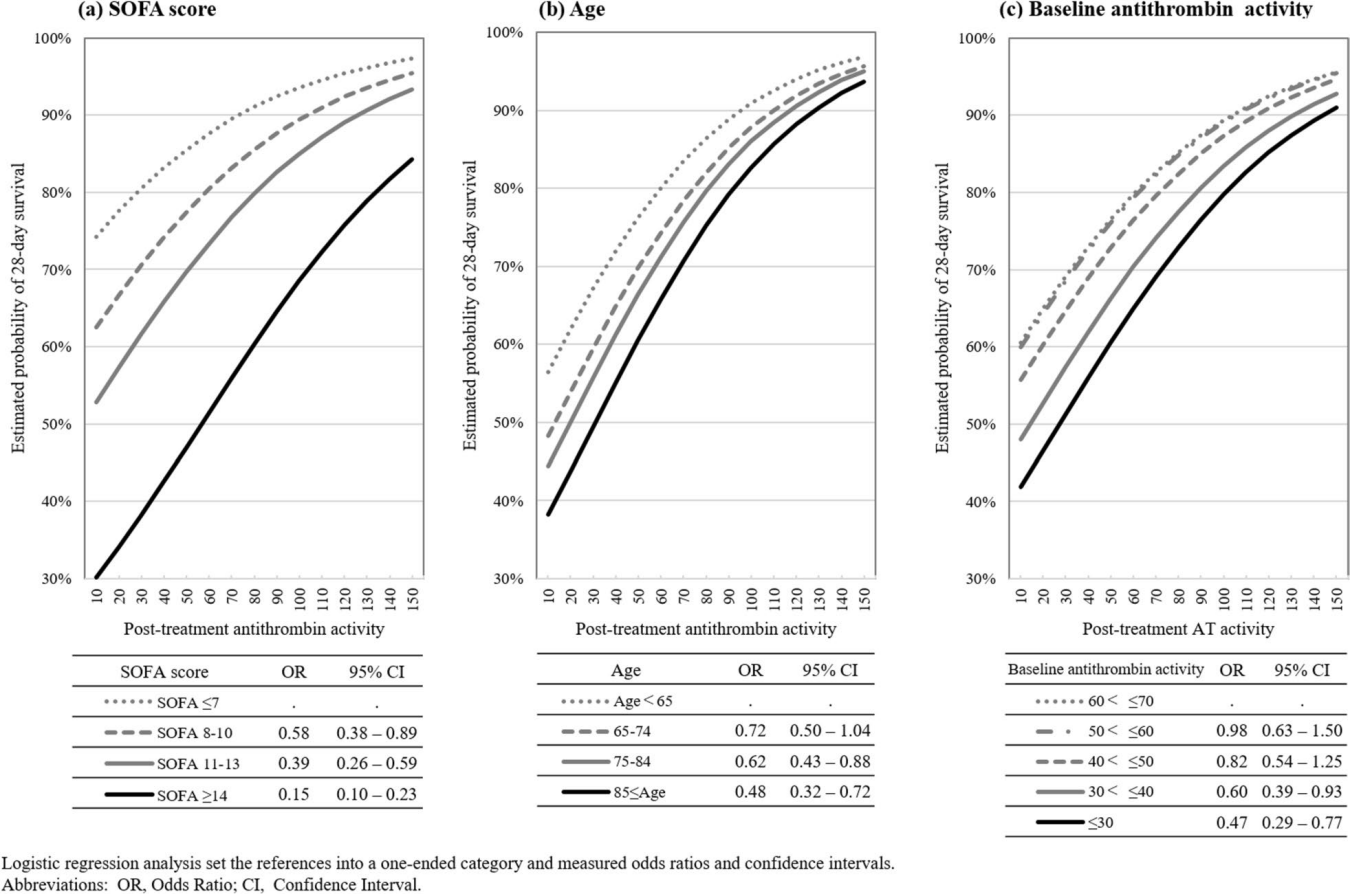
Estimated probability of 28-day survival rate for post-treatment antithrombin activity based on SOFA score, patients’ age, and baseline antithrombin activity. Logistic regression analysis revealed a significant association of the post-treatment antithrombin activity classified by the levels of SOFA score (**a**), patients’ age (**b**), and baseline antithrombin activity (**c**) to 28-day survival rate (*P* < 0.001, respectively). In each analysis, patients were divided into four to five groups based on their score, age, and activity. The 28-day survival rates were higher when the SOFA score was lower, the age was younger, and the baseline antithrombin activity was higher

Logistic regression analysis demonstrated a significant association between post-treatment antithrombin activity and 28-day mortality when patients were categorized based on the levels of baseline SOFA score (a), age (b), and baseline antithrombin activity (c) (*P* < 0.0001, respectively) (Fig. 4), and AUC of post-treatment antithrombin activity based on the baseline SOFA score category increased to 0.688 (Suppl. Figure 2). The OR for 28-day survival was also different depending on the baseline SOFA score (Suppl. Tab. 2). In each analysis, patients were categorized into four to five groups based on their scores, age, and antithrombin activity. The association between post-treatment antithrombin activity and the 28-day survival rate showed significant differences among the groups, with the most pronounced difference observed in the grouping based on SOFA scores.

In this study, bleeding was observed in 94 (5.22%) of 1800 patients. Of these, 85 (4.72%) were serious bleeds, and 14 (0.78%) were considered possibly related to antithrombin supplementation.

## Discussion

The mechanism of decreased antithrombin activity in sepsis-associated DIC is not fully determined, but three potential mechanisms are reported [13]. First, since the physiological role of antithrombin is to neutralize thrombin and other coagulation factors, antithrombin is consumed in patients with activated coagulation [14]. Second, hepatic antithrombin synthesis is suppressed during sepsis, and antithrombin activity decreases gradually following the decreased production [15]. Third, along with vascular injury, circulating antithrombin can leak out rapidly from the intravascular space with increased capillary permeability [16]. These and other factors are involved in the decreased antithrombin activity in sepsis-associated DIC, and the mean activity at the baseline was below 50% in this study which was consistent with the reports from others [11, 17].

As reported previously, post-treatment activity showed superior performance in predicting mortality among the baseline-, post-, and delta antithrombin activity (the difference between posttreatment activity and baseline activity) [10]. The strength of the present study is the ability to determine a relationship between survival and post-treatment antithrombin activity using a continuous scale. A relatively large number of subjects allowed this approach and provided a better understanding of the detailed relationship between antithrombin activity and survival. Consequently, the survival rate decreased more prominently at lower post-treatment antithrombin activity, and a similar trend was recognized in severe cases including patients with sustained DIC, higher SOFA scores, older age, and lower baseline antithrombin activity. This is likely because, in addition to the consumption by activated coagulation, endothelial damage and suppressed synthesis accelerated the decrease in more severe cases. Since the lower antithrombin activity indicates poorer outcomes and is reportedly associated with the enhanced treatment effect of antithrombin [18], the relationship between antithrombin activity and treatment effects should be considered in future studies. Another notable finding in this study is the substantial difference in estimated survival between the early DIC recovery group and the sustained DIC group. This discrepancy was attributed to the relatively low AUC of post-treatment antithrombin activity for 28-day survival. Similar patterns were also observed in the categorization based on baseline SOFA score, age, and baseline antithrombin activity, highlighting the importance of calculating estimated survival based on each specific category. The gaps between the categories were more pronounced when antithrombin activity was low, which can be attributed to the complex mechanisms underlying the decrease in antithrombin. The lower antithrombin activity levels are likely influenced by the increased permeability following endothelial damage, leading to larger discrepancies at the lower activity range [16, 19]. The effect of heparin was not evaluated in the present study, but the concomitant administration of heparin might consume antithrombin.

In the present study, we initially anticipated the decreased antithrombin activity represents the best performance in predicting outcomes since it comprehensively reflects consumption, suppressed production, vascular leakage, and the response to the supplementation. However, post-treatment activity showed the best performance. Therefore, we assume that outcomes are not determined by the mentioned factors but are also affected by the achieved antithrombin activity after antithrombin substitution. The observation that higher post-treatment antithrombin activity is associated with better survival was repeatedly reported [11, 17, 20], and it suggested the idea of aiming the target activity-oriented dose settings. Although the current study results cannot alone support this concept or provide the target dose. However, since the survival rate did not plateau until post-treatment activity reached 150%, and the risk of bleeding was reasonably low, we assume 1500 U/day for three days of supplementation might not be sufficient, and a higher dose is expected. However, it should be cautioned that antithrombin supplementation was avoided for patients with active bleeding or bleeding tendencies, which could have contributed to the low incidence of bleeding. In the previous observational study, we compared the treatment effects of high-dose antithrombin (3,000 IU/day) and low-dose (1,500 IU/day) for 3 days and reported better survival in the high-dose group (77.1% versus 56.4%, *P* = 0.010). In that study, the baseline antithrombin activity was less than 40% and unchanged between the groups, and the post-treatment activity exceeded 100% in the high-dose group, while the activity was approximately 70% in the low-dose group. Furthermore, a logistic regression analysis revealed that antithrombin of 3,000 IU/day was significantly correlated with improved survival (OR, 2.419; *P* = 0.025) [21]. The current study demonstrated a potential link between post-treatment antithrombin activity exceeding 80% and improved survival, implying the potential impact of target-oriented antithrombin administration. However, it's important to note that the antithrombin dose remained fixed in this study, and the hypothesis should be investigated in a study where antithrombin doses are adjusted individually. The effect of high-dose antithrombin should be examined in future prospective randomized controlled trials. At that time, it is important to remember the KyberSept trial’s flaw [22]. After the treatment with extremely high-dose antithrombin (30,000 IU in total over 4 days), antithrombin activity reached approximately 180%, but the survival benefit was not obtained because the trial included patients without coagulation disorder. In the post hoc analysis in patients with coagulation disorder and without concomitant use of heparin, the trend toward better survival was observed (absolute reduction in mortality: 14.6%, *P* = 0.02) [23]. Thus, antithrombin supplementation should be applied only to cases with coagulation disorder and low antithrombin activity in sepsis [9, 17]. With respect to the effect of heparin on antithrombin substitution, the mortality rates in the cases treated with antithrombin without heparin and in the co-administration with heparin cases were 15.8% and 18.0%, respectively. Although the mortality rate was lower in the cases treated without heparin, there was no significant difference (*P* = 0.353). Furthermore, heparin use was not identified as a significant factor that influenced the outcome in the univariate logistic regression analysis (*P* = 0.348), indicating that the effect of heparin was not ascertained in the present study. However, since only 56 cases were treated with antithrombin with heparin, the background of those patients might not be consistent with others, and the amount of antithrombin was far less compared to that used in the KyberSept trial, the effects of concomitant use of heparin are still uncertain. Meanwhile, the effect of heparin on antithrombin activity was not evaluated in the present study, and the concomitant administration of heparin might consume antithrombin.

Recently, the International Society on Thrombosis and Haemostasis/DIC Scientific and Standardization Committee reported antithrombin activity is considered to be the clinically available endothelial damage marker in patients with sepsis-associated DIC [13]. Although previous reports have repeatedly presented the usefulness of antithrombin activity as a prognostic marker [7, 10], the predictive performance of post-treatment antithrombin activity for estimating survival in this study remained at an AUC of 0.639. Therefore, we calculated AUC depending on the baseline SOFA score category, and it rose to 0.688 (Suppl. Figure 2), which is considered clinically valuable.

This study has several limitations. First, since this is a post-marketing survey, the supplementation dose of antithrombin was not fixed. Although most of the patients were treated with 1,500 IU/day of antithrombin for three days, and there was no difference in supplementation doses between survivors and non-survivors, the dose should be controlled in the next study. Second, the baseline antithrombin activity was 70% or less in all the patients, and the patients who showed over 70% of antithrombin activity were not examined in this study. Third, the rate of missing value of post-treatment antithrombin activity was relatively high. The data closest to the post-treatment day replaced 11.7% of the post-treatment value. Although the antithrombin activity did not change rapidly, this might affect the result.

## Conclusion

Serial measurements of antithrombin activity can estimate the prognosis in sepsis-associated DIC. However, estimated survival rates can vary significantly based on the disease severity and should be interpreted differently depending on factors such as baseline SOFA score, age, and baseline antithrombin activity. These variables play a critical role in determining patient outcomes and should be considered when assessing the prognosis. Furthermore, evaluating antithrombin activity after substitution therapy can assist in assessing treatment effectiveness and guide potential adjustments in anticoagulant therapy. Given the relatively low bleeding risk associated with antithrombin substitution, higher doses may be important for patients with high mortality risk and low antithrombin activity. Further studies are needed to confirm this consideration.

### Supplementary Information

**Additional file 1: Supplement figure 1.** Selection of patients with sepsis-associated DIC and antithrombin activity of 70% or less. The data from the patients with sepsis-associated DIC with antithrombin activity of 70% or less and treated with antithrombin concentrate were analyzed. Regarding antithrombin activity and JAAM DIC score, when the data are absent either before or after antithrombin administration, these data are categorized as'missing data'.

**Additional file 2:  Supplement table 1.** Estimated probability of 28-day survival rate for post-treatment antithrombin activity. The values of the logistic regression curves shown in Figure 3 are listed.

**Additional file 3:** **Supplement figure 2 and Supplement table 2.** Receiver operating characteristic curves of the antithrombin activities for 28-day survival calculated depending on the baseline SOFA score. A predictive model was constructed using logistic regression, and its performance was evaluated using Receiver operating characteristic (ROC) analysis. The formula for the predictive score of post-treatment antithrombin activity weighted by the odds ratio of the baseline SOFA score is as follows; If baseline SOFA score ≤7, then 1.018*Post-treatment AT activity + 1 If baseline SOFA score is 8-10, then 1.018*Post-treatment AT activity + 0.58. If baseline SOFA score is 11-13, then 1.018*Post-treatment AT activity + 0.39. If baseline SOFA score ≥14, then 1.018*Post-treatment AT activity + 0.15.

## Data Availability

None.

## Abbreviations

DIC: Disseminated intravascular coagulation
SOFA: Sequential organ failure assessment
ROC: Receiver operating characteristic
AUC: Area under the curve
OR: Odds ratio
CI: Confidence interval
