# Supplementary figures and images for: The antithrombin activity recovery after substitution therapy is associated with improved 28-day mortality in patients with sepsis-associated disseminated intravascular coagulation
Source: Thromb J. 2023 Nov 2;21:112. doi: 10.1186/s12959-023-00556-6 (PMC10621171; doi:10.1186/s12959-023-00556-6)

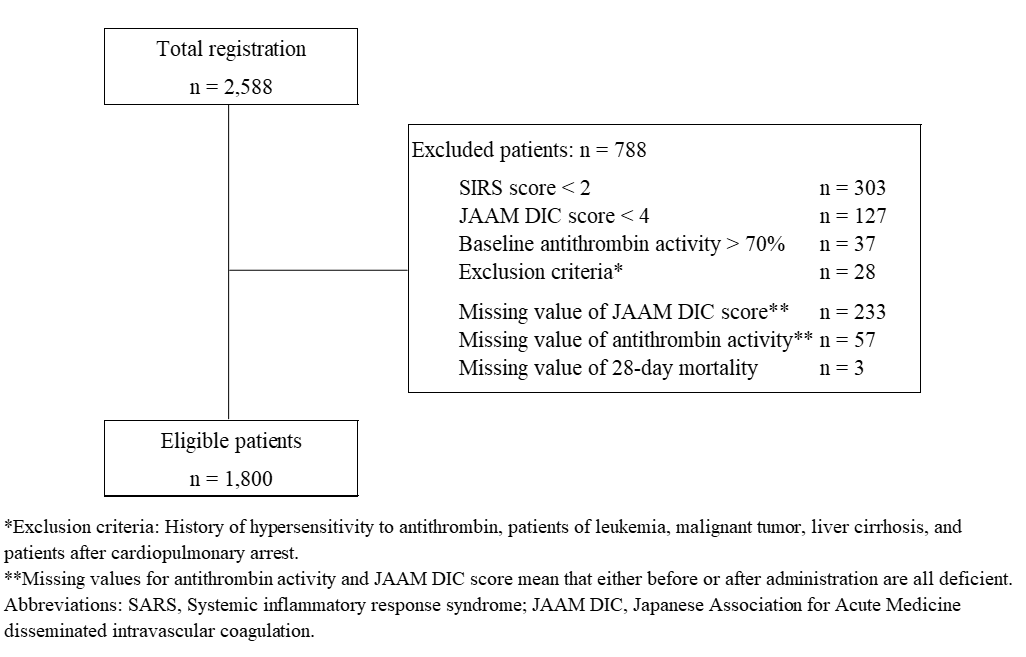

Supplement: Supplementary file 1 — Additional file 1: Supplement figure 1. Selection of patients with sepsis-associated DIC and antithrombin activity of 70% or less. The data from the patients with sepsis-associated DIC with antithrombin activity of 70% or less and treated with antithrombin concentrate were analyzed. Regarding antithrombin activity and JAAM DIC score, when the data are absent either before or after antithrombin administration, these data are categorized as'missing data'. [file 12959_2023_556_MOESM1_ESM.tif]

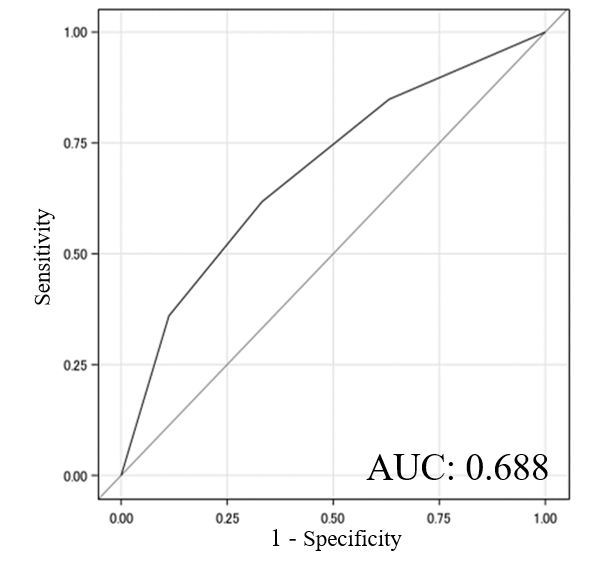

Supplement: Supplementary file 3 — Additional file 3: Supplement figure 2 and Supplement table 2. Receiver operating characteristic curves of the antithrombin activities for 28-day survival calculated depending on the baseline SOFA score. A predictive model was constructed using logistic regression, and its performance was evaluated using Receiver operating characteristic (ROC) analysis. The formula for the predictive score of post-treatment antithrombin activity weighted by the odds ratio of the baseline SOFA score is as follows; If baseline SOFA score ≤7, then 1.018*Post-treatment AT activity + 1 If baseline SOFA score is 8-10, then 1.018*Post-treatment AT activity + 0.58. If baseline SOFA score is 11-13, then 1.018*Post-treatment AT activity + 0.39. If baseline SOFA score ≥14, then 1.018*Post-treatment AT activity + 0.15. [file 12959_2023_556_MOESM3_ESM.zip › Supplement figure2R1.tif]
